# PAR-1 Expression in Chronic Subdural Hematoma: Potential Association with Vascular Permeability

**DOI:** 10.1177/2689288X251383714

**Published:** 2025-10-06

**Authors:** Wataru Shimohigoshi, Hajime Takase, Hiromichi Iwashita, Takashi Kawasaki, Yusuke Kobayashi, Ryosuke Takagi, Takefumi Higashijima, So Ozaki, Shuto Fushimi, Yuya Miyata, Katsumi Sakata, Tetsuya Yamamoto

**Affiliations:** ^1^ Department of Neurosurgery, Yokohama City University Medical Center, Yokohama, Japan.; ^2^ Department of Neurosurgery, Yokohama City University Graduate School of Medicine, Yokohama, Japan.; ^3^ Department of Neurosurgery, Yokosuka General Medical Center, Yokosuka, Japan.; ^4^ Departments of Radiology and Neurology, Massachusetts General Hospital, Harvard Medical School, Charlestown, Massachusetts, USA.; ^5^ YCU Center for Novel and Exploratory Clinical Trials (Y-NEXT), Yokohama City University Hospital, Yokohama, Japan.; ^6^ Department of Pathology Saitama Medical University, Saitama, Japan.; ^7^ YCU Co-Creation Innovation Center, Yokohama City University, Yokohama, Japan.

**Keywords:** chronic subdural hematoma, PAR-1, protease-activated receptor

## Abstract

Chronic subdural hematoma (CSDH) is a common neurosurgical disease in the elderly, characterized by inflammation, neovascularization, and increased vascular permeability. Although protease-activated receptor-1 (PAR-1) is known to regulate vascular permeability and is implicated in chronic inflammatory diseases, its role in CSDH remains unclear. In this exploratory study, we investigated PAR-1 expression in the dura mater and outer membrane of patients with CSDH compared with controls. Age- and sex-matched cases (six CSDH, five controls) were selected for analysis. Immunohistochemistry for PAR-1 and zonula occludens-1 (ZO-1), along with mRNA expression analysis, were performed. Histologically, the outer membrane of CSDH exhibited cellular clustering and strong PAR-1 immunoreactivity in vascular structures, whereas the dura mater from both groups showed no significant PAR-1 staining. ZO-1 expression was preserved in the vasculature of the outer membrane in CSDH and the dura mater of both groups. mRNA analysis revealed a trend toward higher PAR-1 and lower ZO-1 expression in CSDH, though not statistically significant. The group effect (*p* = 0.24, analysis of covariance [ANCOVA] *t*-test) represents the adjusted difference in ZO-1 expression between CSDH and control groups after accounting for PAR-1 levels. The main effect of PAR-1 (*p* = 0.15, ANCOVA *t*-test) reflects the overall association between PAR-1 and ZO-1 expression across samples. This study provides the first evidence of PAR-1 expression in the outer membrane of CSDH, suggesting a role in promoting local vascular hyperpermeability. These findings highlight PAR-1 as a possible biomarker and therapeutic target in CSDH. Further studies with larger cohorts and quantitative analyses are warranted to clarify the molecular mechanisms underlying vascular dysfunction in CSDH.

## Introduction

Chronic subdural hematoma (CSDH) is a disease that primarily occurs in elderly individuals following minor head trauma, leading to a variety of clinical symptoms such as headache, cognitive impairment, and hemiparesis.^1–3^ Previously, CSDH was traditionally regarded as a condition with a favorable prognosis. Recent studies, however, have reported that CSDH could impact on long-term outcomes and mortality.^4,5^ Post-traumatic inflammatory response, neovascularization, and increased vascular permeability are considered to be important risk factors for its development and recurrence. Although endothelial inflammation mediated by the vascular endothelial growth factor–NF-κB pathway in the hematoma’s outer membrane may contribute to hematoma expansion and recurrence,^6^ the broader molecular mechanisms underlying CSDH remain largely unclear.^7–9^

Protease-activated receptors (PARs) are G protein-coupled receptors involved in coagulation and inflammation, and are expressed in both immune cells and vascular endothelial cells.^10,11^ Among these, PAR-1 plays an important role in regulating vascular endothelial permeability and has been highlighted as a potential therapeutic target in cancer and inflammatory disorders.^12^ Although inflammation is considered to be a major contributor in the pathogenesis of CSDH, the role of PAR-1 in CSDH has not been previously studied. Therefore, in this study, we exploratorily investigated PAR-1 expression in normal dura mater, CSDH-associated dura mater, and the outer membrane of patients with CSDH. Our aim was to clarify the involvement of PAR-1 in CSDH and to evaluate its potential as a novel biomarker and therapeutic target.

In addition to immunohistochemical analysis, we also examined the mRNA expression of PAR-1 and zonula occludens-1 (ZO-1) in the dura mater. Rather than pursuing broad transcriptomic profiling, this approach was intended to validate and extend our histological findings by quantitatively assessing PAR-1 and ZO-1 expression at the RNA level. By comparing expression patterns in age- and sex-matched dura samples from patients with CSDH and controls, we aimed to evaluate transcriptional alterations associated with vascular barrier dysfunction and to explore the regulatory relationship between these two genes in the context of CSDH pathology.

## Materials and Methods

This study was based on the secondary use of clinical samples and data originally collected under a prior study (IRB approval F220700004) at Yokohama City University, and was conducted with separate IRB approval for this analysis (F241200004). Written informed consent for secondary use was obtained from all prospectively enrolled participants. Immunohistochemistry (IHC) for PAR-1 and ZO-1 was designated as the primary analysis, with RNA sequencing of dura mater specimens serving as a complementary molecular validation. Age- and sex-matched cases were selected for both IHC and RNA analyses. Dura mater samples were collected from both control and CSDH groups, and outer membrane samples were additionally obtained from patients with CSDH. Due to the limited availability of clinical samples, it was not feasible to use the same tissue set for both assays. As a result, IHC and RNA analyses were performed on separate cohorts. All procedures followed standard protocols, as detailed in the Supplementary Data.

## Results

Among the 11 patients enrolled in the primary analysis (5 controls and 6 with CSDH), age and sex were matched between the groups. The control group included four males, while the CSDH group included five males (*p* = 0.89). The mean ages were 68.0 ± 8.5 years and 74.8 ± 11.4 years for the control and CSDH groups, respectively (*p* = 0.31). The control group consisted of four patients with Parkinson’s disease and one with idiopathic normal pressure hydrocephalus (details shown in Supplementary Tables S1 and S2). There were no significant differences in hematological data between the two groups except for alanine aminotransferase. All patients in the CSDH group underwent burr hole drainage, and there were no recurrences during the follow-up period.

For the secondary RNA analysis, eight control patients and five patients with CSDH were included. The control cohort comprised seven patients with Parkinson’s disease and one with meningioma. The control group included five males, and all patients in the CSDH group were male (*p* = 0.23). Because this study was based on the secondary use of clinical samples collected in a prior study, the availability of specimens was limited. In particular, only a limited amount of clinical tissue could be obtained from patients with CSDH or Parkinson’s disease due to the constraints of the surgical procedures performed. Consequently, sex matching was prioritized during sample selection, but age matching could not be achieved. The mean ages were 65.8 ± 6.5 years and 76.6 ± 4.8 years for the control and CSDH groups, respectively (*p* < 0.01). Detailed demographic and clinical characteristics are provided in Supplementary Table S3.

### Hematoxylin and eosin staining

**FIG. 1.**
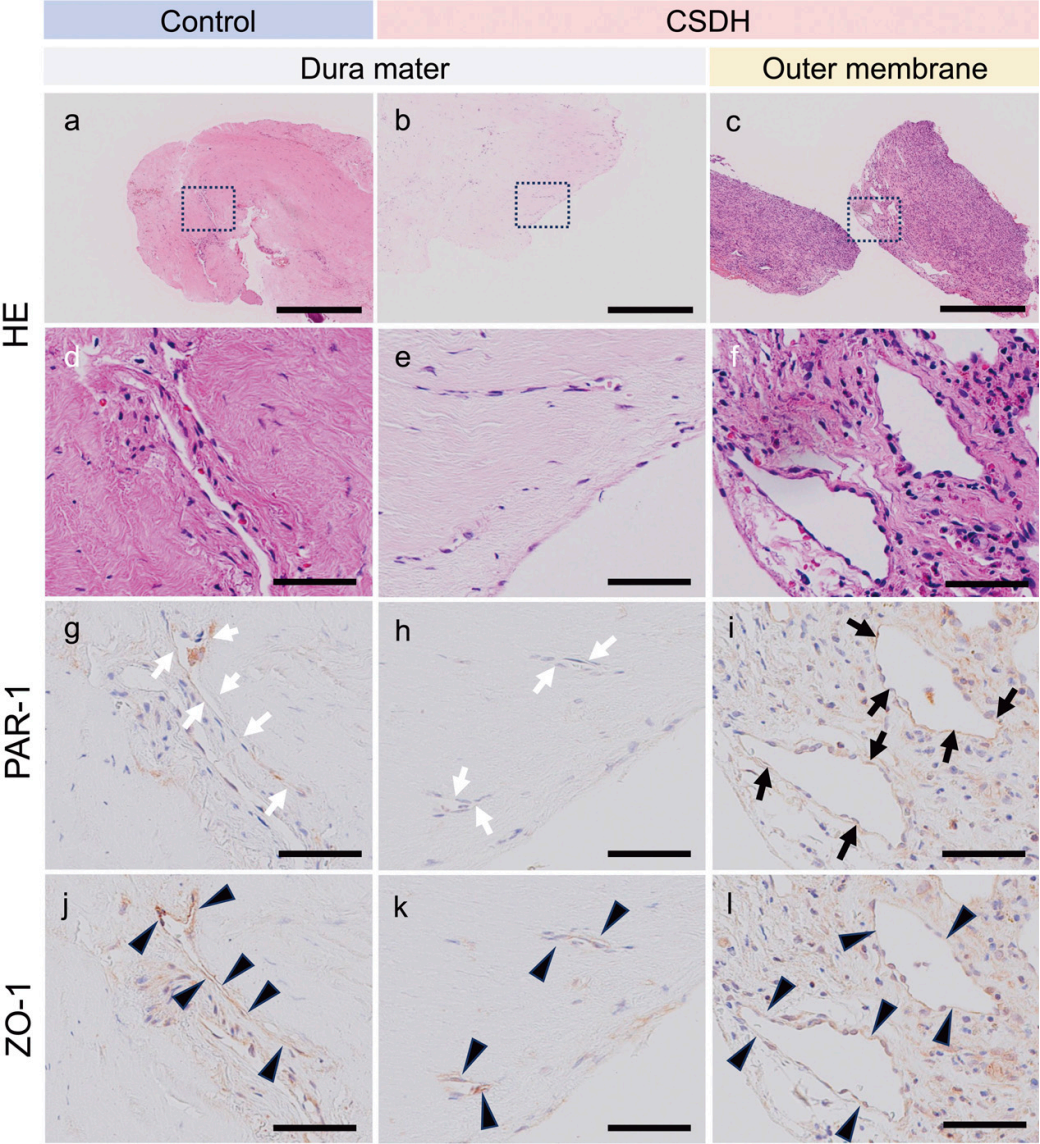
Representative histological and immunohistochemical findings in the dura mater and outer membrane of control and chronic subdural hematoma (CSDH) groups. **(a–f)** Hematoxylin and eosin (HE) staining images. **(a, d)** Dura mater from a control subject. **(b, e)** Dura mater from a patient with CSDH. **(c, f)** Outer membrane from a patient with CSDH. Low-magnification views (2.5×) are shown in **(a–c)** (scale bar, 500 μm), and corresponding high-magnification views (20×) in **(d–f)** (scale bar, 50 μm). The outer membrane **(f)** contains abundant microvessels compared with the dura mater in both groups **(d, e)**. **(g–i)** Immunohistochemistry (IHC) for protease-activated receptor-1 (PAR-1; scale bar, 50 μm). PAR-1 expression was specifically observed in cells presumed to be endothelial cells within the outer membrane in the CSDH group (i, black arrows), while no notable PAR-1 staining was detected in cells lining dural vessels in either group (g, h, white arrows). **(j–l)** IHC for zonula occludens-1 (ZO-1; scale bar, 50 μm). ZO-1 expression was detected in the microvascular endothelial cells of the dura mater from both control **(j)** and CSDH **(k)** groups, as well as in the outer membrane (l), where it was localized to endothelial cells (black arrowheads).

There were no obvious structural differences between the dura mater of the control and CSDH groups by hematoxylin and eosin (HE) staining (Fig. 1a, b, d, e). In contrast, the outer membrane of CSDH showed cellular clustering and increased neovascularization, as already reported (Fig. 1c, f).

### PAR-1 staining

No obvious positive signal of PAR-1 was observed in the vasculature of the dura mater in either the control or CSDH groups (Fig. 1g, h). In contrast, strong signals were seen in the inner cells of the vasculature, suspecting endothelial cells, in the outer membrane of CSDH (Fig. 1i).

### ZO-1 staining

ZO-1 expression was detected in the dural vasculature in both the control and CSDH groups, as well as in the outer membrane of the CSDH group (Fig. 1j–l).

### RNA analysis

**FIG. 2.**
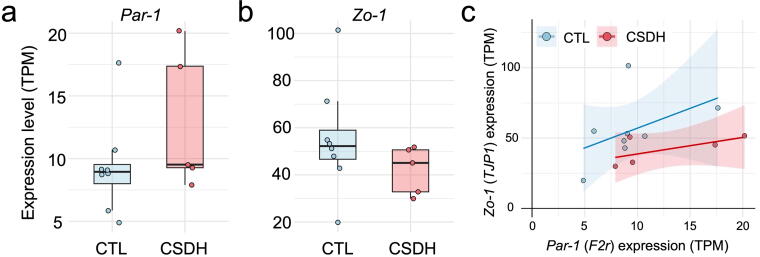
RNA expression analyses. **(a, b)** Group-wise comparison of PAR-1 and ZO-1 expression. Box plots show the expression levels of PAR-1 and ZO-1 in control (CTL) and chronic subdural hematoma (CSDH) groups. Individual data points are overlaid as jittered dots. PAR-1 expression was modestly higher in the CSDH group without reaching statistical significance (*p* = 0.22, Wilcoxon rank-sum test). Similarly, ZO-1 expression tended to be lower in the CSDH group compared with CTL, but this difference was not statistically significant (*p* = 0.22, Wilcoxon rank-sum test). **(c)** ZO-1 expression plotted against PAR-1 expression with group-wise linear regression with 95% confidence intervals is shown for each group. Scatter plots represent individual data points for each group. No significant interaction was found between group and PAR-1 expression (*p* = 0.54, interaction *F*-test), indicating that the relationship between PAR-1 and ZO-1 did not differ between groups. The group effect (*p* = 0.24, ANCOVA *t*-test) represents the difference in ZO-1 expression between CTL and CSDH groups after adjusting for PAR-1 levels. The main effect of PAR-1 (*p* = 0.15, ANCOVA *t*-test) reflects the overall association between PAR-1 and ZO-1 expression regardless of group. PAR-1, protease-activated receptor-1; ZO-1, zonula occludens-1; ANCOVA, analysis of covariance.

To examine PAR-1 and ZO-1 mRNA expression levels in the dura mater, we first compared control (CTL) and CSDH groups using nonparametric Wilcoxon rank-sum tests. PAR-1 expression was modestly higher in the CSDH group compared with the CTL group, although the difference was not statistically significant (Fig. 2a; *p* = 0.22). Similarly, ZO-1 expression tended to be lower in the CSDH group than in the CTL group, but this difference also did not reach statistical significance (Fig. 2b; *p* = 0.22). These findings suggested potential group-associated trends in gene expression, albeit not conclusive due to the small sample size (CTL: *n* = 8, CSDH: *n* = 5). To further explore the relationship between PAR-1 and ZO-1 expression, we performed group-wise linear regression and analysis of covariance (ANCOVA), with PAR-1 expression included as a covariate (Fig. 2c). No statistically significant interaction was found between group and PAR-1 expression (*p* = 0.54, interaction *F*-test), indicating that the relationship between PAR-1 and ZO-1 did not differ between CTL and CSDH groups. After adjusting for PAR-1, the difference in ZO-1 expression between groups was not statistically significant (group effect: *p* = 0.24, ANCOVA *t*-test), and the main effect of PAR-1 also did not reach significance (*p* = 0.15, ANCOVA *t*-test). Nonetheless, both effects showed modest trends. These results collectively suggest a possible association between vascular permeability-related genes in CSDH, warranting further validation in larger cohorts.

## Discussion

### Overview

In this exploratory study, we investigated the expression of PAR-1 in the dura mater and outer membrane of patients with CSDH. Through immunohistochemical and RNA-based analyses, we demonstrated for the first time that PAR-1 is strongly expressed in the vasculature of the outer membrane, but not in the dura mater, of CSDH cases. In contrast, ZO-1, a key tight junction protein, was consistently expressed across all groups. Although our RNA expression analysis did not reveal statistically significant differences between groups, we observed trends toward increased PAR-1 and decreased ZO-1 expression in CSDH. These findings suggest a possible association between PAR-1 signaling and vascular permeability in the CSDH microenvironment, supporting the hypothesis that PAR-1 may contribute to local vascular dysfunction in this disease.

### HE staining

Numerous microvessels were observed in the outer membrane of CSDH. This result is thought to reflect inflammation-induced neovascularization in the outer membrane of CSDH consistent with previous studies.^13,14^ In the dura mater of the CSDH group, qualitative histological assessment revealed that blood vessels appeared smaller in diameter and fewer in number compared with those in the control group, suggesting possible vascular damage. This observation implies ongoing vascular remodeling and chronic inflammatory changes. However, these findings are based on visual inspection and should be interpreted with caution.

### Protease-activated receptor-1

PAR-1 has been reported to increase vascular permeability in other tissues.^10,15^ In our results, strong PAR-1 signals were observed in the inner layer of blood vessels within the outer membrane of CSDH, but not in the dura mater of either the control or CSDH groups. This finding suggests that PAR-1 expression in the outer membrane may contribute to vascular hyperpermeability, consistent with previous reports indicating increased permeability in neovessels involved in the expansion and recurrence of CSDH.^16,17^

### Zonula occludens-1

ZO-1, a representative component of tight junctions, plays a critical role in maintaining endothelial barrier function. Previous studies have reported that activation of PAR-1 reduces ZO-1 expression, thereby leading to increased permeability.^18,19^ In this study, ZO-1 expression was observed in the blood vessels of both the outer membrane and the dura mater (in both control and CSDH groups), and appeared to be maintained despite the presence of PAR-1. This may reflect an early stage in the development of vascular hyperpermeability.

### RNA analysis

We hypothesized that increased expression of PAR-1 would downregulate ZO-1 and thereby increase vascular permeability in the dura mater, particularly in the context of CSDH. To test this, we assessed both immunohistochemical and mRNA expression levels of PAR-1 and ZO-1 in dura mater samples (CTL: *n* = 8, CSDH: *n* = 5; see Supplementary Fig. S1). However, our exploratory analyses did not demonstrate a statistically significant inverse association between PAR-1 and ZO-1 expression. ANCOVA revealed that neither the group effect (*p* = 0.24) nor the main effect of PAR-1 expression (*p* = 0.15) reached statistical significance, although both showed modest trends. While inconclusive, these trends may suggest that PAR-1 contributes to vascular barrier modulation in a context-dependent manner. Given that PAR-1 can act through diverse downstream pathways and various cell types, including macrophages and fibroblasts in the outer membrane, it is plausible that its influence on endothelial tight junction proteins such as ZO-1 is indirect or requires the presence of additional inflammatory mediators.^20,21^ Despite the limitations of this study, most notably its small sample size, the combined histological and transcriptional data provide preliminary insight into the vascular and molecular environment of the CSDH-associated dura. These findings warrant further investigation in larger, well-characterized cohorts, ideally incorporating additional markers of vascular integrity and barrier function.

### Limitations and future perspectives

This study has several limitations. First, the small sample size due to its exploratory nature limited the statistical power of our findings. Second, the immunohistochemical evaluation was qualitative rather than quantitative, and, as mentioned above, no experiments were conducted to directly assess functional changes in vascular permeability. Third, the control group mainly included patients with Parkinson’s disease, which may have affected dural pathology to some extent. However, obtaining truly healthy human dural samples is practically unfeasible, and this represents an inherent limitation of the study. In addition, due to the limited availability of clinical specimens, IHC and RNA sequencing were performed on different cohorts, which may limit the direct comparability between these two datasets. Furthermore, age matching could not be achieved in the RNA cohort, as CSDH is generally a disease of the elderly, making it difficult to obtain appropriately aged control dura samples. Therefore, caution is warranted when generalizing the present findings to the broader population, particularly to neurologically healthy individuals.

Despite these limitations, we successfully demonstrated for the first time the expression of PAR-1 in vascular cells within the outer membrane of CSDH, most likely endothelial cells based on their location. Given the well-established role of PAR-1 in modulating vascular endothelial permeability, our findings raise the possibility that PAR-1 contributes to local barrier dysfunction and potentially to the progression or recurrence of CSDH.

Future studies should incorporate larger, well-characterized cohorts with appropriate neurologically healthy controls. In addition, Vorapaxar, a clinically approved PAR-1 inhibitor, is currently used as an antiplatelet agent. However, its potential application in bleeding disorders such as CSDH remains challenging. Quantitative IHC, mechanistic investigations using *in vitro* and *in vivo* models, and functional assays focused on endothelial and inflammatory responses will be critical in elucidating the role of PAR-1 in CSDH pathogenesis. Ultimately, this line of research could lead to the identification of PAR-1 as a novel therapeutic target for modulating vascular dysfunction in CSDH and related neuroinflammatory conditions.

## Transparency, Rigor, and Reproducibility Statement

This study was conducted as an exploratory observational analysis. The sample size was determined to be the minimum necessary to establish age- and sex-matched cohorts, while avoiding excessive enrollment inconsistent with an exploratory design. Blinding was implemented during immunohistochemical evaluation to reduce observer bias. As this was a clinical observational study, no randomization was applied. All data and analytical codes used in this study are available upon reasonable request from the corresponding author.

## Supplementary Material

Supplementary Data

Supplementary Table S1

Supplementary Table S2

Supplementary Table S3

## Abbreviations Used

ANCOVA: analysis of covariance
ALT: alanine aminotransferase
CSDH: chronic subdural hematoma
CTL: control
HE: hematoxylin and eosin
IHC: immunohistochemistry
NF-κB: nuclear factor kappa B
PAR-1: protease-activated receptor-1
VEGF: vascular endothelial growth factor
ZO-1: zonula occludens-1
