# Supplementary material for: PAR-1 Expression in Chronic Subdural Hematoma: Potential Association with Vascular Permeability
Source: Neurotrauma Rep. 2025 Oct 6;6(1):956–62. doi: 10.1177/2689288X251383714 (PMC12549182; doi:10.1177/2689288X251383714)
Supplement: Supplementary Data [file 2689288x251383714_suppl_data.docx]

**Supplementary materials**

***Study participants and inclusion/exclusion criteria***

The study included all patients, regardless of gender, aged 18 years or older at the time of surgery; the CSDH group enrolled patients diagnosed and operated on for chronic subdural hematoma (CSDH), if they had a preoperative head CT. Patients were not asked whether they had a recurrence. However, patients with a history of blood coagulation abnormalities, patients with acute subdural hematoma or strong clinical suspicion of subdural abscess, and patients with suspected CSDH caused by cerebrospinal fluid hypovolemia were excluded. In the control group, patients who did not have CSDH and required resection of normal dura mater or dura mater including normal areas were included. Specifically, patients who required dural resection for functional surgery and patients with lesions, such as meningiomas, that required a large dural resection that included normal areas were included. Note that patients with meningitis were excluded from the study.

***Sample collection and clinical data***

Dura mater samples were obtained from both the CSDH and control groups, and outer membrane samples were additionally collected from patients in the CSDH group. All samples used in this study were derived from the prospectively enrolled subset of the prior study, for which written informed consent for secondary use had been obtained. To minimize the sample size in this exploratory study, age- and sex-matched cases were selectively included: 6 CSDH patients and 5 control patients. Clinical data including age, sex, comorbidities, surgical procedure, histopathological findings, and medication use were collected. In the CSDH group, preoperative CT findings and recurrence within 3 months postoperatively were also recorded.

***Immunohistochemistry (IHC)***

Collected small tissues were fixed in formalin and paraffin-embedded for histological analysis. Sections (4 μm) were prepared for staining. To evaluate the expression of PAR-1 and ZO-1 in tissue samples from both groups, IHC was performed using hematoxylin and eosin (HE) as a counterstain. HE staining was performed at the Department of Pathology, Yokohama City University Hospital, and IHC for PAR-1 and ZO-1 was performed at GenoStaff Co., Ltd. (Tokyo, Japan). All stained sections were evaluated by a pathologist with expertise in histopathology. Tissue sections were deparaffinized and subjected to antigen retrieval by microwave heating in EDTA buffer (pH 9). Endogenous peroxidase activity was blocked with 0.3% hydrogen peroxide/methanol. Nonspecific binding was blocked using G-Block, followed by avidin/biotin blocking. Sections were incubated with mouse monoclonal anti-PAR-1 antibody (Abcam, ab233741; 2 μg/mL) or rabbit polyclonal anti-ZO-1 antibody (Invitrogen, 40-2200, 0.4 μg/mL) at 4°C. As negative controls, mouse IgG1 (Dako, X0931) for PAR-1 and rabbit IgG (Vector, I-1000) for ZO-1 were used. After washing, sections were incubated with biotinylated secondary antibodies and peroxidase-conjugated streptavidin and stained with DAB and hydrogen peroxide.

***RNA analysis***

Fresh surgical dura mater specimens were promptly washed with saline, blotted to remove excess moisture, and snap-frozen at –80°C. Total RNA was extracted using the QIAzol method with ethanol-based phase separation. RNA sequencing was performed using a ribosomal RNA depletion protocol. Libraries were prepared and sequenced on an Illumina platform to generate approximately 20 million paired-end reads per sample (150 bp × 2). Transcript quantification was performed using standard pipelines, and TPM (transcripts per million) values were used for expression analysis. Note that the number of samples used for RNA sequencing differs from that of the immunohistochemistry cohort. In particular, only a limited amount of clinical tissue could be obtained from patients in the CSDH group and from control patients with Parkinson’s disease due to the constraints of the surgical procedures performed. Because this study was based on the secondary use of clinical samples originally collected in a prior study, the available pool of specimens was limited. As a result, sample selection for RNA analysis was performed independently from the IHC cohort. In selecting the RNA cohort, sex matching was prioritized; however, age matching could not be achieved. Consequently, the cohorts used for IHC and RNA analysis were not identical.

***Statistical analysis***

All statistical analyses and visualizations were conducted using R (version 4.5.0) on macOS Sequoia 15.1. Data visualization was performed with the ggplot2 package (v3.5.2), while data wrangling and transformation were handled using dplyr (v1.1.4) and tidyr (v1.3.1). Annotated box plots and group-wise statistical comparisons were generated with ggpubr (v0.6.0). Linear modeling and analysis of covariance (ANCOVA) were performed using base functions in the stats package, and natural spline regression was implemented via the splines package. Linear models were fitted using the lm() function, interaction terms were evaluated by F-tests using the anova() function, and Wilcoxon rank-sum tests for pairwise group comparisons were performed using ggpubr::stat_compare_means().

**Supplementary figure**

**
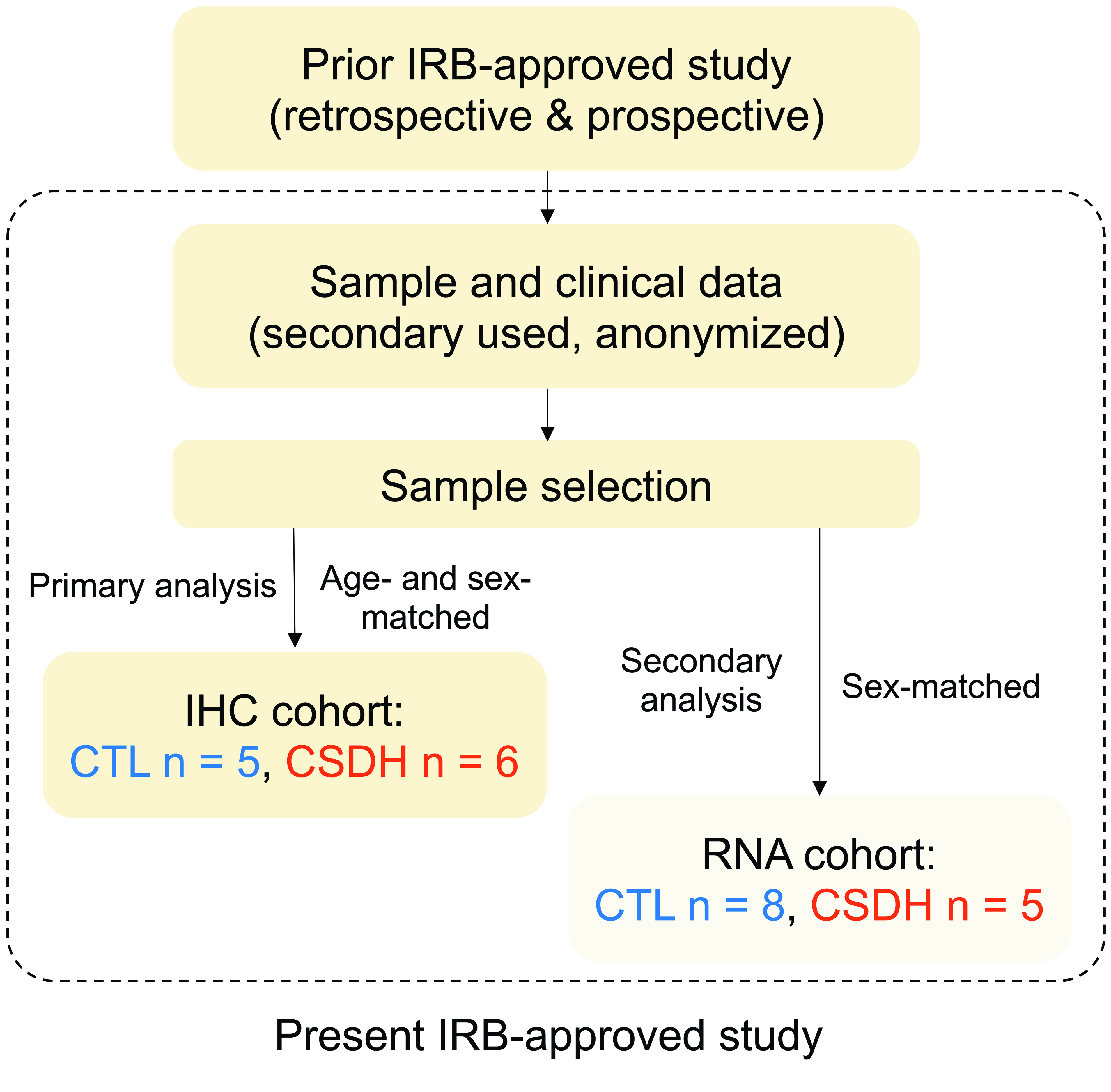
Figure S1. Overview of study design and sample selection.**
Samples and clinical data were secondarily utilized from a prior IRB-approved study, which included both retrospective and prospective components. Age- and sex-matched patients were selected for the primary analysis using immunohistochemistry (IHC cohort; CTL n = 5, CSDH n = 6). RNA expression analysis via RNA sequencing was conducted as a secondary analysis (RNA cohort; CTL n = 8, CSDH n = 5). All data were anonymized prior to analysis.
