# Supplementary material for: PAR-1 Expression in Chronic Subdural Hematoma: Potential Association with Vascular Permeability
Source: Neurotrauma Rep. 2025 Oct 6;6(1):956–62. doi: 10.1177/2689288X251383714 (PMC12549182; doi:10.1177/2689288X251383714)
Supplement: Supplementary Table S1 [file 2689288x251383714_suppl_tables1.docx]

**Table S1.** Demographics and clinical characteristics of patients in the control and chronic subdural hematoma groups.

|  | Group | |  |
| --- | --- | --- | --- |
|  | Control | CSDH | Univariate, *P-value* |
| Number of patients | 5 | 6 |  |
|  | Parkinson 4 |  |  |
|  | iNPH 1 |  |  |
| Sex, (%Male) | 4 (80.0) | 5 (83.3) | 0.89 |
| Age, year (SD) | 68.0 (8.5) | 74.8 (11.4) | 0.31 |
| Height, cm (SD) | 165.2 (14.1) | 161.1 (7.0) | 0.93 |
| Weight, kg (SD) | 66.8 (8.7) | 62.2 (9.5) | 0.78 |
| BMI (SD) | 24.5 (2.7) | 24.1 (3.4) | 0.93 |
| HT (%) | 3 (75.0) | 3 (60.0) | 0.74 |
| DM (%) | 2 (50.0) | 1 (20.0) | 0.39 |
| DL (%) | 2 (50.0) | 1 (20.0) | 0.39 |
| Antithrombotic drugs (%) | 1 (20.0) | 0 (0) | 0.33 |
| Antihypertensive drugs (%) | 3 (75.0) | 3 (60.0) | 0.74 |
| Statin (%) | 1 (20.0) | 1 (16.7) | 0.86 |
| Herbal drugs (%) | 1 (25.0) | 2（33.3） | 0.62 |
| Levodopa preparations (%) | 4(80.0) | 0 (0) | 0.006 |
| Recurrence (%) | 0 (0) | 0 (0) | NA |
| WBC (10^3/uL) | 6.5 (1.1) | 8.2 (5.1) | 1.00 |
| RBC (10^6/uL) | 4.5 (0.3) | 4.3 (0.7) | 0.78 |
| HGB (g/dL) | 13.4 (1.4) | 13.7 (2.2) | 0.52 |
| HCT (%) | 41.9 (3.6) | 41.6 (6.2) | 0.78 |
| PLT (10^3/uL) | 258.2 (80.5) | 218.3 (77.6) | 0.41 |
| AST (U/L) | 21.8 (8.7) | 27.5 (13.9) | 0.41 |
| ALT (U/L) | 10.8 (6.1) | 23.0 (10.5) | 0.04 |
| LDH (U/L) | 184.2 (15.4) | 217.8 (111.8) | 1.00 |
| ADP (U/L) | 90.8 (19.7) | 128.2 (76.5) | 0.32 |
| γ-GTP (U/L) | 30.6 (16.4) | 56.0 (82.6) | 1.00 |
| TP (g/dL) | 7.1 (0.5) | 7.0 (0.5) | 0.78 |
| Alb (g/dL) | 4.4 (0.1) | 4.2 (0.5) | 0.45 |
| BUN (mg/dL) | 19.4 (6.4) | 24.7 (14.8) | 0.71 |
| Cre (mg/dL) | 0.8 (0.1) | 1.0 (0.2) | 0.10 |
| T-Bil (mg/dL) | 0.7 (0.3) | 0.9 (0.2) | 0.76 |
| Na (mmol/L) | 141.8 (2.6) | 139.0 (5.5) | 0.40 |
| K (mmol/L) | 4.2 (0.3) | 4.5 (0.6) | 0.52 |
| Cl (mmol/L) | 106.6 (3.0) | 103.8 (7.2) | 0.58 |
| Glu (mg/dL) | 126.0 (49.5) | 122.0 (43.9) | 1.00 |
