# Supplementary material for: PAR-1 Expression in Chronic Subdural Hematoma: Potential Association with Vascular Permeability
Source: Neurotrauma Rep. 2025 Oct 6;6(1):956–62. doi: 10.1177/2689288X251383714 (PMC12549182; doi:10.1177/2689288X251383714)
Supplement: Supplementary Table S2 [file 2689288x251383714_suppl_tables2.docx]

**Table S2.** Raw data of patient demographics and clinical characteristics in this study.

| Patients | 1 | 2 | 3 | 4 | 5 | 6 | 7 | 8 | 9 | 10 | 11 |
| --- | --- | --- | --- | --- | --- | --- | --- | --- | --- | --- | --- |
| Group | CSDH | CSDH | CSDH | CSDH | CSDH | CSDH | Control | Control | Control | Control | Control |
| Main disease | CSDH | CSDH | CSDH | CSDH | CSDH | CSDH | PD | iNPH | PD | PD | PD |
| Sex | M | M | M | M | F | M | M | M | F | M | M |
| Age | 73 | 82 | 71 | 81 | 84 | 53 | 54 | 76 | 74 | 67 | 67 |
| Height/cm | 173.0 | 155.7 | 175.0 | 159.0 | 136.8 | 166.9 | 173.9 | 164.5 | 155.6 | 162.6 | 169.6 |
| Weight/kg | 54.0 | 65.3 | 71.7 | 70.2 | 47.2 | 64.2 | 70.1 | 63.0 | 65.2 | 56.2 | 79.6 |
| BMI | 18.0 | 26.9 | 23.4 | 27.8 | 25.2 | 23.0 | 23.2 | 23.3 | 26.9 | 21.3 | 27.7 |
| Other disease | HT | DM | HT | HT | HT | Cervical spine injury | HU | HT |  | HT | HT |
|  | Meningioma | HU |  |  | CHF |  | Appendicitis | Gastrointestinal ulcer |  | DM | DM |
|  | Reflux esophagitis |  |  |  | HL |  |  |  |  | Gastrointestinal ulcer | HL |
|  |  |  |  |  | HU |  |  |  |  |  |  |
| WBC, 10^3/uL | 5.0 | 8.0 | 18.4 | 6.7 | 5.9 | 5.1 | 7.8 | 5.7 | 5.2 | 6.6 | 7.3 |
| RBC, 10^6/uL | 4.52 | 4.09 | 4.84 | 4.36 | 3.19 | 5.06 | 5.07 | 4.43 | 4.36 | 4.30 | 4.52 |
| HGB, g/dL | 13.0 | 14.5 | 15.1 | 14.3 | 9.6 | 15.9 | 15.3 | 11.5 | 13.5 | 12.9 | 13.6 |
| HCT, % | 40.1 | 43.4 | 46.0 | 42.7 | 29.9 | 47.3 | 46.9 | 37.6 | 40.8 | 40.0 | 44.1 |
| PLT, 10^3/uL | 204 | 131 | 332 | 219 | 144 | 280 | 393 | 227 | 184 | 264 | 223 |
| AST, U/L | 17 | 22 | 41 | 19 | 49 | 17 | 37 | 18 | 17 | 16 | 21 |
| ALT, U/L | 17 | 16 | 19 | 22 | 44 | 20 | 20 | 11 | 4 | 7 | 12 |
| LDH, U/L | 162 | 201 | 442 | 149 | 195 | 158 | 183 | 191 | 188 | 200 | 159 |
| ADP, U/L | 122 | 132 | 91 | 66 | 276 | 82 | 80 | 79 | 114 | 110 | 71 |
| γ-GTP, U/L | 9 | 22 | 44 | 19 | 223 | 19 | 36 | 16 | 28 | 17 | 56 |
| TP, g/dL | 6.9 | 7.5 | 7.4 | 7.2 | 6.1 | 6.9 | 7.9 | 6.8 | 6.8 | 6.9 | 7.0 |
| Alb, g/dL | 4.4 | 4.1 | 4.5 | 4.2 | 3.3 | 4.5 | 4.5 | 4.5 | 4.3 | 4.3 | 4.4 |
| BUN, mg/dL | 30 | 22 | 13 | 14 | 52 | 17 | 14 | 24 | 12 | 27 | 20 |
| Cre, mg/dL | 1.13 | 1.18 | 0.71 | 0.85 | 1.01 | 0.91 | 0.9 | 0.89 | 0.62 | 0.61 | 0.85 |
| T-Bil, mg/dL | 0.7 | 0.8 | 1.2 | 0.6 |  | 1.0 | 1.0 | 0.4 | 0.8 |  |  |
| Na, mmol/L | 142 | 142 | 128 | 139 | 142 | 141 | 141 | 138 | 142 | 145 | 143 |
| K, mmol/L | 4.8 | 4.1 | 3.8 | 4.3 | 5.5 | 4.6 | 3.9 | 4.6 | 4.4 | 4.2 | 4.1 |
| Cl, mmol/L | 105 | 106 | 91 | 105 | 113 | 103 | 103 | 104 | 107 | 110 | 109 |
| Ca, mg/dL |  | 9.4 | 9.1 | 9.5 |  | 9.5 |  | 9.5 |  |  |  |
| Mg, mg/dL |  |  | 1.6 |  |  |  |  |  |  |  |  |
| Glu, mg/dL |  | 110 | 187 | 97 |  | 94 |  | 176 |  | 125 | 77 |
| CRP, mg/dL |  | 0.4 | 0.2 | 0.1 | 1.4 | 0.2 |  | 0.1 |  |  | 0.1 |
| Antithrombotic drugs | 0 | 0 | 0 | 0 | 1 | 0 | 0 | 0 | 0 | 0 | 0 |
| Antihypertensive drugs | 1 | 0 | 0 | 1 | 1 | 0 | 0 | 0 | 0 | 1 | 1 |
| Statin | 0 | 0 | 0 | 0 | 1 | 0 | 0 | 0 | 0 | 0 | 1 |
| Herbal drug | 1 | 1 | 0 | 0 | 0 | 0 | 0 | 0 | 0 | 1 | 0 |
| Levodopa preparations | 0 | 0 | 0 | 0 | 0 | 0 | 1 | 0 | 1 | 1 | 1 |
| Drug details | Amlodipine | Metformin |  | Amlodipine | Amlodipine |  | L-DOPA /Carbidopa | Acamprosate | L-DOPA /Carbidopa | L-DOPA /Carbidopa | L-DOPA /Carbidopa |
|  | Candesartan | Sitagliptin |  |  | Atorvastatin |  | Febuxostat | Tamsulosin | Levothyroxine | Amlodipine | Telmisartan |
|  | Lansoprazole | Allopurinol |  |  | Aspirin |  | Alprazolam | Mirabegron | Methimazole | Esomeprazole | Teneligliptin |
|  | Goreisan | Goreisan |  |  | Sacubitril Valsartan |  | Zonisamide | Amlodipine | Mirabegron | Sitagliptin | Rosuvastatin |
|  |  |  |  |  | Carbocisteine |  |  | Irbesartan | Domperidone | Lubiprostone | Istradefylline |
|  |  |  |  |  | Mecobalamin |  |  | Doxazosin | Zonisamide | Tiapride | Linaclotide |
|  |  |  |  |  | Ezetimibe |  |  | Atorvastatin | Safinamide | Risperidone |  |
|  |  |  |  |  | Spironolactone |  |  |  | Opicapone | Sennosides |  |
|  |  |  |  |  | Lansoprazole |  |  |  | Zolpidem | Yokukansan |  |
