# Supplementary material for: PAR-1 Expression in Chronic Subdural Hematoma: Potential Association with Vascular Permeability
Source: Neurotrauma Rep. 2025 Oct 6;6(1):956–62. doi: 10.1177/2689288X251383714 (PMC12549182; doi:10.1177/2689288X251383714)
Supplement: Supplementary Table S3 [file 2689288x251383714_suppl_tables3.docx]

**Table S3.** Demographics and clinical characteristics of the cohorts used for RNA analysis in the control and CSDH groups.

|  | Group | |  |
| --- | --- | --- | --- |
|  | Control | CSDH | Univariate, *P-value* |
| Number of patients | 8 | 5 |  |
|  | Parkinson 7 |  |  |
|  | meningioma 1 |  |  |
| Sex, (%Male) | 5 (62.5) | 5 (100) | 0.23 |
| Age, year (SD) | 65.8 (6.5) | 76.6 (4.8) | < 0.01 |
| Height, cm (SD) | 160.1 (15.0) | 166.5 (8.6) | 0.59 |
| Weight, kg (SD) | 55.4 (10.2) | 65.2 (7.0) | 0.10 |
| BMI (SD) | 21.6 (2.6) | 23.2 (3.9) | 0.28 |
| HT (%) | 2 (25.0) | 3 (60.0) | 0.29 |
| DM (%) | 1 (12.5) | 1 (20.0) | 0.39 |
| DL (%) | 0 (0) | 0 (0) | NA |
| Recurrence (%) | 0 (0) | 0 (0) | NA |
